# Adapting Radiology Magnetic Resonance Safety Workflows to Magnetic Resonance Imaging Simulation in Radiation Oncology

**DOI:** 10.7759/cureus.110376

**Published:** 2026-06-06

**Authors:** Rachel A Sabol, Charlotte S Chan, Nicolas D Prionas, Christina Calvin, Luis Pelayo, Haley Randolph, Sherman Lim, Craig Devincent, Michael Ohliger, Javier Villanueva-Meyer, Jessica Scholey, Lisa Singer

**Affiliations:** 1 Department of Radiation Oncology, University of California San Francisco, San Francisco, USA; 2 Department of Radiology, University of California San Francisco, San Francisco, USA

**Keywords:** interdisciplinary collaboration education, magnetic resonance (mr), mri safety, mr simulation, quality improvement and patient safety

## Abstract

Objective: With the use of MRI simulation and MRI-linacs, magnetic resonance imaging (MRI) is becoming increasingly integrated into radiation oncology (RO) departments, requiring new safety considerations. While American College of Radiology guidelines describe MRI safety broadly, there is limited published experience in adapting these workflows to RO-specific settings and equipment, such as MRI simulators. This quality improvement study evaluated the impact of MRI safety interventions in RO at a single institution.

Methods: In an effort to improve safety surrounding the use of a 3-T MRI simulator in RO, three plan-do-study-act (PDSA) cycles were implemented at a single institution. Cycle 1 implemented a two-screen functional workflow used in radiology, adapted for the RO setting; cycle 2 implemented department-wide education; and cycle 3 introduced a visual aid to assist with screening. The MRI schedule was retrospectively reviewed for safety endpoints during each cycle. Endpoints evaluated included the number of same-day cancellations, the number of patients identified at the initial screen as having an implant, and safety events (SEs).

Results: The percentages of MRI simulations that were same-day cancellations during PDSA cycles 1-3 were 6.6%, 10.4%, and 7.4%, respectively (p = 0.51). The number of patients identified at the initial screen as potentially having an implant that needs review by the MRI safety team, representing early identification of high-risk patients, was zero, zero, and three across the three PDSA cycles, respectively. There were no SEs during the study.

Conclusion: An MRI safety workflow developed in radiology was successfully implemented in RO. The number of patients successfully screened as high-risk at the initial screen increased after repeat education. Although overall cancellation rates did not significantly change, these findings suggest that repeated targeted education and visual reference tools may improve early detection of potentially unsafe implants. There were no SEs during the study period. Further improvements in early identification of high-risk patients could decrease same-day cancellations and increase the efficiency of MRI simulation, a limited departmental resource.

## Introduction

Radiation therapy (RT) is a major component of cancer care delivered to over half of cancer patients during the course of their disease [1]. A primary goal of RT is to maximize the dose to the target while minimizing exposure to adjacent organs at risk (OARs). To achieve this end, precise target delineation is required and has been made possible by advancements in imaging and image-guided RT [2]. The introduction of computed tomography (CT) simulation into radiation oncology (RO) has enhanced the precision of target delineation and dose distribution to the planning target volumes. However, in many cases, CT is limited by inadequate soft-tissue contrast for target and OAR delineation, leading to the development and implementation of magnetic resonance imaging (MRI) simulators [3,4].

Compared to CT, MRI provides superior soft-tissue contrast, resulting in improved target delineation in the context of RT [2,3]. For example, a study evaluating prostate target volumes drawn using CT compared to MRI found that prostate volume was defined on average 1.4 times larger on CT compared to on MRI [5]. Furthermore, the precise anatomical definition provided by MRI at the prostate-rectal interface enables higher doses to be more confidently delivered to smaller target volumes [6]. By improving the resolution of soft tissue contrast for tumors and surrounding critical OARs, MRI-based delineation has been shown to reduce toxicity [7,8]. MRI confers the additional advantage of high-contrast imaging without exposure to ionizing radiation. The advantages of MRI have therefore led to the increasing utilization of MRI technology in RT with the development and adaptation of MRI simulators and with devices combining MRI scanners with linear accelerators for treatment delivery, or MRI-linacs.

Due to the advantages of MRI and the development of MRI simulators and MRI-linacs, MRI scanners are increasingly housed directly in RO departments [2,9,10]. Integrating MRI into RO departments introduces new safety considerations for patients and staff specific to MRI due to the strong electromagnetic fields produced by MRI scanners [9,11]. For patients with metal devices or implants, the static magnetic field may cause ferromagnetic objects to become projectiles or to experience torque, leading to safety incidents. In addition, the radiofrequency field may cause heating. The time-varying gradient fields may stimulate peripheral nerves and compromise the function of active implants [12,13]. Although it is estimated that 10%-20% of general MRI patients have metal devices or implants, a retrospective study reports that over 70% of cancer patients were treated via implanted port catheters, which may be MRI conditional or unsafe [11,14]. Furthermore, one RO department found that over 40% of MRI simulation patients had metal within the imaging field [15]. Considering that a significant proportion of cancer patients have metal implants and foreign bodies related and unrelated to their cancer diagnosis, such as surgical clips, ports, surgical hardware after orthopedic surgery, ventriculoperitoneal shunts, and other devices, MRI safety represents a crucial area of concern for RO departments as technological advancements are implemented.

In radiology departments, screening and appropriate risk assessment have been shown to effectively mitigate adverse clinical events among MRI patients with active metal implants [16]. As MRI technology becomes integrated into the RT workflow, RO departments must adopt similar screening workflows to identify and triage patients with potentially MRI-unsafe implants prior to scheduling MRI simulations. Foreign bodies that are not identified until the point of care during the MRI exam disrupt clinical workflow, same-day cancellations, and inefficient allocation of limited departmental resources. More importantly, an effective systematic MRI safety screening workflow minimizes risk to patients and staff by preventing the missed identification of MRI-unsafe implants.

The American College of Radiology (ACR) has MRI safety guidelines that set the standard for safe implementation of MRI [17]. Although these guidelines can be extrapolated to RO, detailed RO-specific guidelines are lacking. The American Association of Physicists in Medicine (AAPM) has two task groups (TGs) that provide some RO-specific MRI safety guidance, TG 284 and TG 334 [18,19]. TG 334 focuses on MRI safety of RT-specific devices, an important consideration for the use of MRI technology in RO, though not as relevant to patient-specific screening workflows that are the focus of our current investigation [18]. TG 284 suggests that in nonemergent scenarios, which would apply to most RO patients undergoing MRI for treatment planning or treatment, patients should undergo at least two screenings for implanted devices and metal foreign bodies [19], consistent with ACR MRI safety guidelines that advise two step screening for all patents [17]. A two-screen MRI safety protocol is important because a second screen has been found to identify up to 34% of implants that were not identified in the first screen [13]. Additionally, personnel training and staff education have been recommended as integral components of establishing MRI safety [9,15,20]. This study describes a single-institution quality improvement (QI) experience that implemented an MRI safety workflow developed by the radiology department in the RO department and included educational interventions. The purpose of this QI effort was to reduce same-day cancellations of MRI simulations and improve MR safety.

## Materials and methods

**Figure 1:**
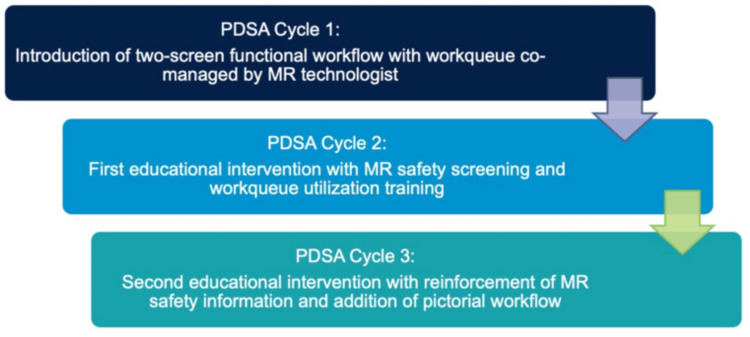
Study overview Visual diagram of the three PDSA cycles during the study associated with the implementation of the MRI safety workflow with MRI simulation WQ to triage high-risk implants. PDSA cycle 1 implemented a two-screen functional workflow that included a WQ, adapted from the diagnostic radiology department at the same institution. PDSA cycle 2 was a quality improvement educational intervention for PCs who perform the first screen. PDSA cycle 3 was a second educational intervention, which included reinforcement of MRI education and a handout to reference at the time of scheduling, walking through the decision tree for the first MRI safety screen PDSA: plan-do-study-act; MRI: magnetic resonance imaging; WQ: workqueue; PCs: practice coordinators; MR: magnetic resonance

To improve MRI safety in RO, three plan-do-study-act (PDSA) cycles were initiated surrounding the use of a 3-T MRI simulator at an academic center. The cycles were implemented from April 18, 2022, to January 19, 2023 (Figure 1). These PDSA cycles included adapting a radiology workflow to the RO department, which included an electronic health record workqueue (WQ), and educational interventions targeted at staff involved in scheduling MR simulations.

MRI safety oversight for the simulator was provided by a multidisciplinary team with members from the departments of radiology and RO, all of whom were required to complete MRI safety training as mandated by institution policy. Endpoints evaluated after each PDSA cycle included the total number of MRI simulations scheduled, the number of same-day cancellations, the number of patients in the WQ (a measure of the number of patients identified at the primary screen as having a high-risk implant), and safety events (SEs) in each PDSA cycle. It was hypothesized that implementing a two-step screening process with the WQ would decrease the number of same-day cancellations, thereby improving efficiency and throughput on a limited departmental resource. Statistical analysis using the chi-square test was performed, and p values ≤0.05 were considered significant.

Interventions

**Figure 2:**
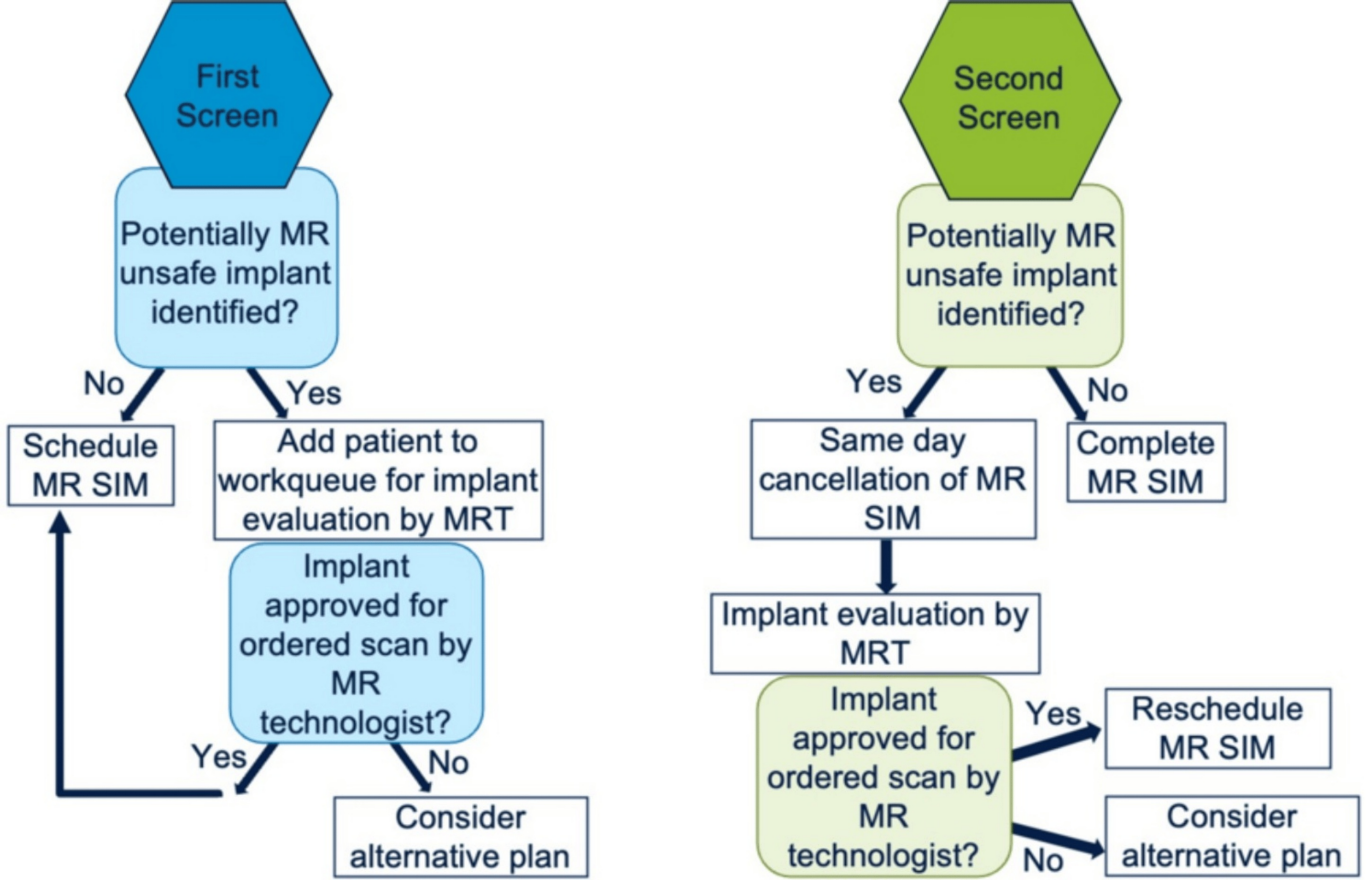
MRI safety two-screen functional workflow with workqueue This diagram outlines the two-screen functional workflow that was implemented. The first screen is completed by the PC at the time of MRI SIM scheduling. If a potentially MRI-unsafe implant is identified at the first screen, the PC triages high-risk patients into a WQ for further evaluation by the MRI safety team. The MRT and MRI safety team co-manages the WQ and investigates if the implant identified as high risk in the first screen is compatible with the ordered MRI simulation. If the implant is deemed compatible with the desired scan, an MRI simulation will be scheduled. If the implant is considered incompatible with the desired scan, the MRT and MRI safety team will notify the clinical team of the need for an alternative imaging plan. The second screen is performed by the MRT at the point of care when the patient arrives for the MRI simulation. If, at the point of care, a potentially MRI unsafe implant or foreign body is identified, the patient would not be able to proceed with MRI that day, and the MRI simulation will be canceled to allow for adequate time for safety evaluation of the implant by the MRT, leading to the same-day cancellation of the MRI simulation and need for either rescheduling of the MRI simulation PC: practice coordinator; MRI: magnetic resonance imaging; SIM: simulation; WQ: workqueue; MRT: magnetic resonance imaging technologist; MR: magnetic resonance

PDSA cycle 1 implemented a two-screen functional workflow, adapted from the diagnostic radiology department at the same institution, which includes a WQ, which is an electronic health record-shared patient task list. The first screen is completed by the practice coordinator (PC) when scheduling the MRI simulation. If an MRI-unsafe or potentially unsafe foreign body or implant is identified on the first screen, the PC triages high-risk patients into a WQ for further evaluation by the MRI safety team. The MRI technologist (MRT) co-manages the WQ and investigates if the implant identified as high-risk in the first screen is compatible with the ordered MRI simulation prior to scheduling. The MRT and RO MRI safety team will determine whether the foreign body or implant is compatible with the desired scan and proceed with scheduling the MRI simulation, or, if incompatible, notify the ordering provider of the need for an alternative imaging plan. A second screen is performed by the MRT at the point of care when the patient arrives for the MRI simulation. If at the point of care a second screen identifies a potentially MRI unsafe implant or foreign body, the patient would not be able to proceed with MRI that day, and the MRI simulation would be canceled to allow for adequate time for safety evaluation of the implant by the MRT and the RO MRI safety team (Figure 2).

**Figure 3:**
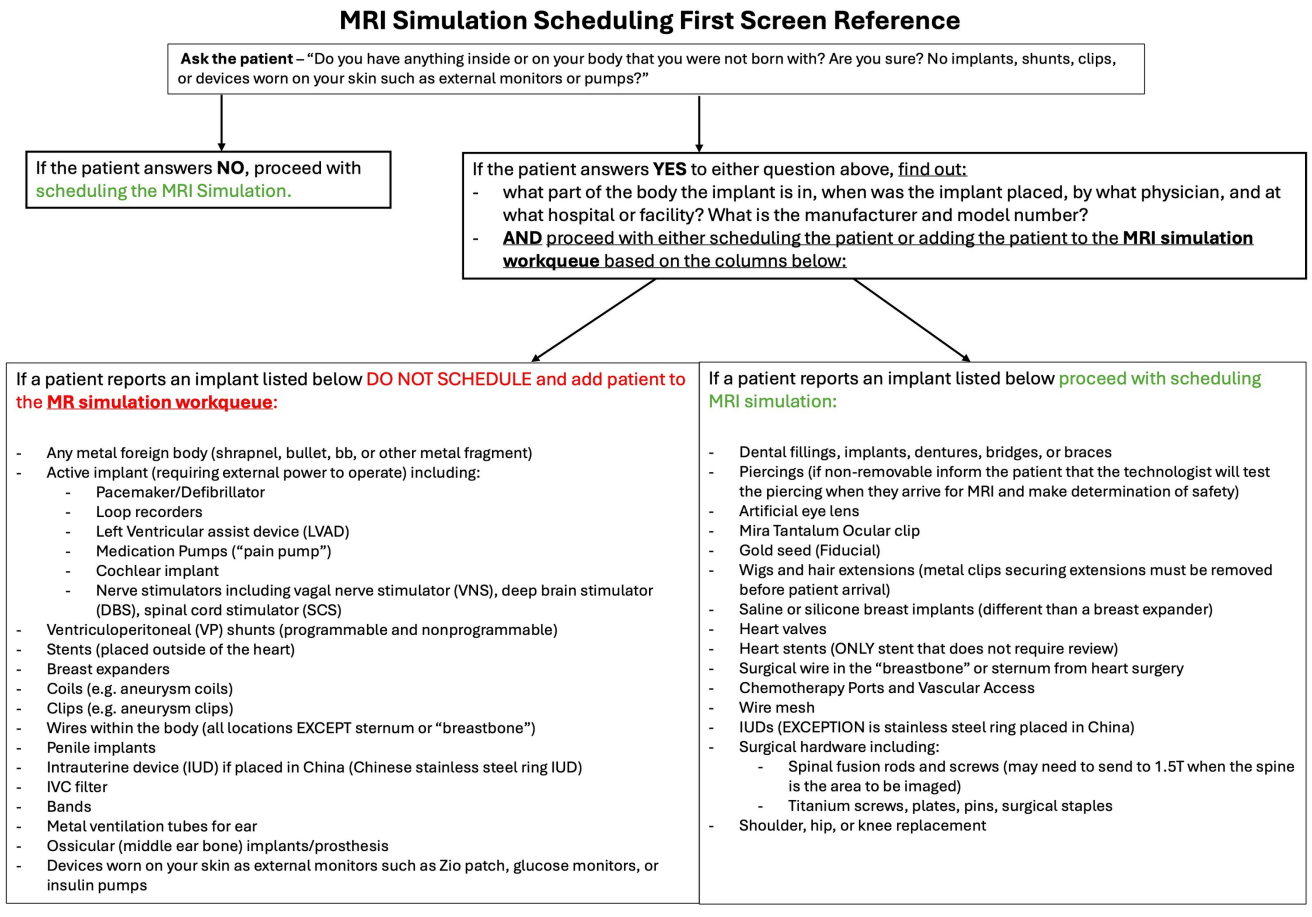
First screen MRI safety reference example This figure presents an example of the visual diagram provided as part of PDSA cycle 3 in the second educational intervention for the first MRI safety screen done at the time of MRI simulation scheduling by the practice coordinators. This diagram and the list of MRI-safe and unsafe implants are continuously reviewed and updated by our institution’s MRI safety committee PDSA: plan-do-study-act; MRI: magnetic resonance imaging

After implementing this WQ into the MRI simulation scheduling workflow, two educational cycles were conducted to improve staff screening for potentially MRI-unsafe implants or foreign bodies during the first screen. The goal of the educational initiatives was to reduce the number of potentially MRI-unsafe implants identified at the second/point-of-care screen, which can lead to same-day cancellations. PDSA cycle 2 was after the first PC educational intervention. PDSA cycle 3 followed a second educational intervention, including reinforcement of MRI education and a handout to reference at the time of scheduling that walks through the decision tree and lists implants to be screened (as shown in Figure 3). This handout is continuously reviewed and updated by our institution’s MRI safety committee.

## Results

**Figure 4:**
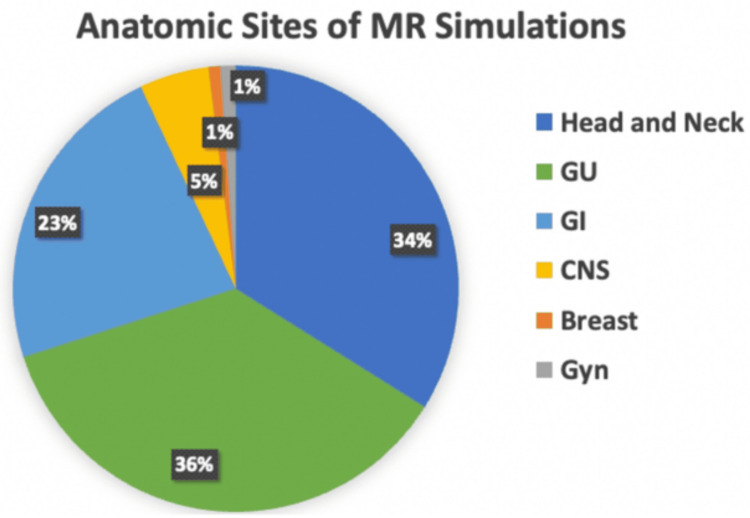
Overview of MRI simulations The relative frequency of MRI simulations ordered across different body/disease sites during the study period is shown MR: magnetic resonance; GU: genitourinary; GI: gastrointestinal; CNS: central nervous system; Gyn: gynecologic; MRI: magnetic resonance imaging

During this study period, 358 MRI simulations were evaluated. Nearly two-thirds (61.7%) of the simulations ordered were for stereotactic body radiation therapy (SBRT), and 38.2% were for intensity-modulated radiation therapy (IMRT). MRI simulations were ordered for treatment of a variety of disease sites, with the most common in this series at our institution being for genitourinary cancer (36%), head and neck (34%), and gastrointestinal (22%) (Figure 4).

PDSA cycle 1 spanned 56 workdays, during which 91 MRI simulations were scheduled, with six same-day cancellations. PDSA cycle 2 spanned 84 days during which 173 MRI simulations were scheduled with 18 same-day cancellations. PDSA cycle 3 spanned 39 workdays and had 94 MRI simulations, with seven same-day cancellations. The percentage of same-day cancellations out of the total scheduled MRIs was 6.6%, 10.4%, and 7.4% in PDSA cycles 1-3, respectively. A chi-square analysis revealed no significant difference in the proportion of canceled vs. completed MRI simulations between cycles (chi-square 1.322, p = 0.51). The same-day cancellation rate for SBRT vs. IMRT treatments was evaluated separately, and no association was found between the number of same-day cancellations per cycle and treatment modality. The number of patients in the WQ during each PDSA cycle, representing successfully screened high-risk patients for further triage by the MRT, was zero, zero, and three, respectively. There were no SEs during the study (Table 1).

**Table 1:** Results across PDSA cycles This table shows the events during each PDSA cycle, including the number of days, number of MRI simulations, number of same-day cancellations, number of patients triaged to the WQ, and number of safety events. There was no significant difference in the same-day cancellations per scheduled MRI simulations across the three PDSA cycles (chi-square 1.322, p = 0.51) PDSA: plan-do-study-act; MRI: magnetic resonance imaging; WQ: workqueue

PDSA	Days in cycle	MRI simulations	Same-day cancellations	Patients in the WQ	Safety events
Cycle 1	56	91	6	0	0
Cycle 2	84	173	18	0	0
Cycle 3	39	94	7	3	0

## Discussion

An MRI safety workflow adapted from diagnostic radiology was successfully implemented in RO for MRI simulations. Repeated MRI safety education improved utilization of the WQ to identify and triage high-risk patients prior to point of care for MRI, which can lead to same-day cancellations. In this study, there was no significant difference in the proportion of same-day cancellations of MRI simulations. No SEs occurred during the study. While this study described the successful adaptation of this workflow from a radiology department to a single RO department, a similar workflow could be applied to other RO departments.

Implementing multiple screens to identify implants is well supported in the literature, by ACR guidelines and AAPM TG 284 [13,19,21]. One study notes that a single-screen process resulted in a high number of "near-misses," with 39% of incidents related to unsafe screening. Implementing a multistep screening process effectively lowered near-misses to 0% [21]. More specifically, a key element of MRI safety training is effective communication through face-to-face instruction supplemented by forms of multimedia [20]. Following these guidelines, our workflow involves both staff education and a visual reference handout as an aid, which can be referenced during screening. In support of our findings, the use of multiple, repeated educational interventions has been shown to be an effective strategy for other institutions implementing similar safety policies [21].

Despite the strengths of this study in successfully adapting a workflow from radiology to RO, no significant reduction in same-day cancellations was seen after implementation of the two-screen workflow with WQ and multiple educational interventions. The lack of improvement in same-day cancellations could be attributed to a small number of MRI simulations in each PDSA cycle, given that this is a single-institution QI study and/or a lag between the implementation of interventions and the impact on reduction in cancellations. Other limitations include PDSA cycle length variation and different patient volume across cycles, which limit comparisons across cycles. Same-day cancellations remain a challenge due to the identification of MRI-unsafe implants on a second screen; however, these implants have been identified using the two-screen system, and no SEs have occurred. Another challenge in MRI safety is that safety information continually changes as annual guidelines are updated and new medical devices are used in patients. The list of potentially MRI-unsafe implants and foreign bodies is routinely reviewed and updated by the institutional MRI Safety Committee, as new implants become available and as the safety profiles of existing implants are better understood. This is critical for safety but poses a challenge and requires ongoing updates and education to be dispersed to all staff involved in the MRI screening workflow. Dedicated time and resources to staff involved in MRI scheduling are important, as MRI safety education is a moving target.

The generalizability of this study is limited in that it takes place at a single institution where the MRI simulator is housed in the RO and staffed by the RO. Individual hospitals may differ in staffing models for MRI simulation. In some departments, radiology may provide safety oversight of MRI simulation, while in other departments, this oversight may be in RO or in a shared model involving both radiology and RO. Regardless of the staffing model, MRI safety is critical. The need for MRI safety education has increased as MRI technology is increasingly integrated into RO departments for MRI simulations. The risks posed by these machines should not be underestimated, and without proper precautions, they could lead to serious harm or even death [19]. Additionally, early identification of potentially unsafe implants allows adequate time to evaluate their safety. Proper safety screening prevents cancellations and ensures efficient use of this limited resource. More importantly, it could prevent treatment delays, which are associated with worse outcomes for cancer patients [22]. Implementing an MRI safety program, therefore, represents an important next step as RO departments strive to deliver safe, state-of-the-art patient care.

## Conclusions

Implementation of a two-step MRI safety screening workflow designed for a diagnostic radiology department was successfully implemented in RO for MRI simulation. Although there was no significant difference in the proportion of same-day cancellations between PDSA cycles, utilization of the WQ increased in PDSA cycle 3 after a repeat educational intervention and the introduction of a visual aid, which is continuously updated by a multidisciplinary MRI safety committee. This highlights improvements in the successful screening and identification of high-risk patients through workflow adaptation and repeat educational interventions. There were no SEs in any PDSA cycle. These data highlight the importance of education and re-education to stakeholders, as MRI technology is implemented directly into RO departments.
